# Supplementary material for: A Membrane with Strong Resistance to Organic and Biological Fouling Using Graphene Oxide and D-Tyrosine as Modifiers
Source: Membranes (Basel). 2022 Apr 29;12(5):486. doi: 10.3390/membranes12050486 (PMC9145901; doi:10.3390/membranes12050486)
Supplement: Supplementary file 1 [file membranes-12-00486-s001.zip › membranes-1626362-supplementary.pdf]

Supplementary Information: A membrane with Strong Resistance to Organic and Biological Fouling Using Graphene Oxide and D-Tyrosine as Modifiers

Jiarui Guo [1,2], Yan Zhang [1,2,*], Fenghua Chen [2] and Yuman Chai [2]

1 Key Laboratory of Water Quality Science and Water Environment Recovery Engineering, Beijing University of Technology, Beijing 100124, China
2 Faculty of Architecture, Civil and Transportation Engineering, Beijing University of Technology, Beijing 100124, China; guojiarui@emails.bjut.edu.cn (J.G.); chenfh@cnpe.cc (F.C.); chaiyuman@163.com (Y.C.)
* Correspondence: yzhang@bjut.edu.cn

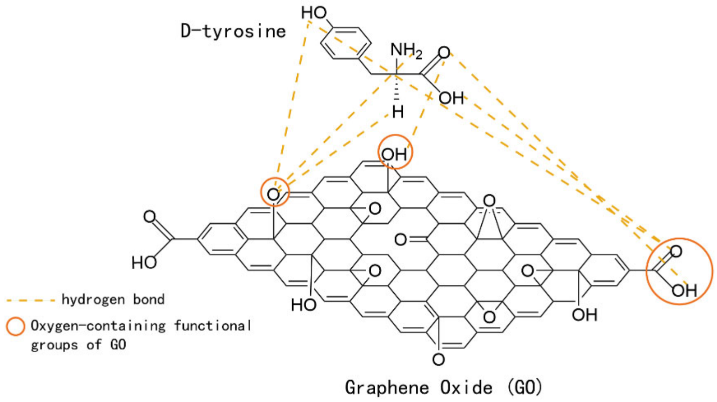

Figure S1. Schematic diagram of the Intermolecular hydrogen bonding between GO and D-tyrosine in the membrane matrix.

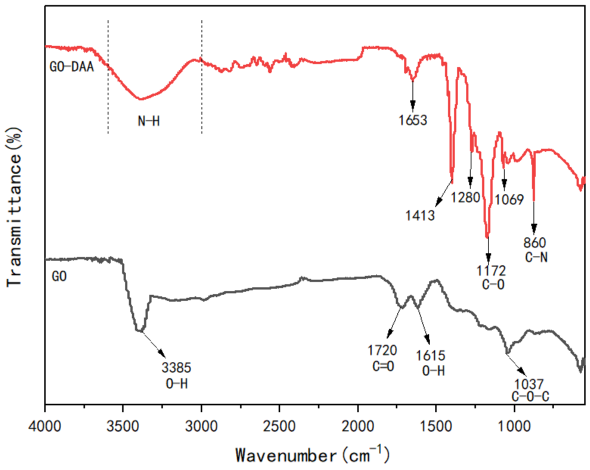

Figure S2. FTIR spectra of GO and GO-DAA.

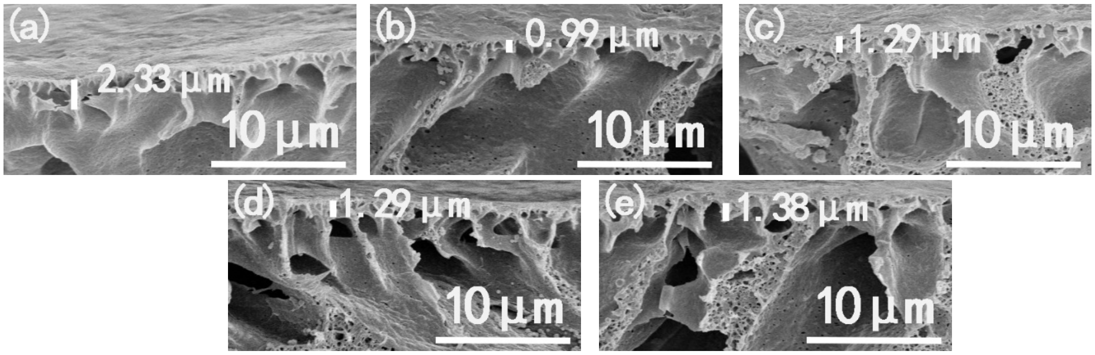

Figure S3. The surface layer thickness of the membranes. (a) PVDF; (b) P-GO; (c) P-GO-50; (d) P-GO-100; (e) P-GO-150.

Table S1. Components in the polymer casting solutions.

Membrane	Substance and concentration				
	DMAc (wt %)	PVP (wt %)	PVDF (wt %)	GO (wt %)	D-tyrosine (mg/L)
PVDF	80	2	18	/	/
P-GO	79.8	2	18	0.2	/
P-GO-50DAA	79.8	2	18	0.2	50
P-GO-100DAA	79.8	2	18	0.2	100
P-GO-150DAA	79.8	2	18	0.2	150

Table S2. Elemental composition of three membranes' surface.

Membrane	Atomic percentage (%)		
	C 1s	O 1s	N 1s
PVDF	83.05	11.11	5.84
P-GO	86.54	8.57	4.89
P-GO-DAA	84.69	9.03	6.28

Table S3. Carbon chemical bond composition of three membranes' surface.

Membrane	Carbon chemical bond (%)			
	C-C	C-F	C=C	C-O/C-N
PVDF	45.04	41.11	8	5.85
P-GO	51.06	30.18	11.87	6.89
P-GO-DAA	41.51	30.99	16.52	10.97

Table S4. Average pore size and porosity of fouled membranes.

Membrane	Average pore size (nm)	Porosity (%)
PVDF	2.9 (±1.6)	16.5 (±1.8)
P-GO	7.1 (±1.8)	40.1 (±2.6)
P-GO-50DAA	9.9 (±2.1)	58.8 (±1.8)
P-GO-100DAA	13.2 (±1.7)	72.0 (±2.3)
P-GO-150DAA	12.3(±1.9)	69.6 (±2.1)

Table S5. Infrared absorption peaks with corresponding chemical bonds.

Absorption peaks (cm^{-1})	chemical bonds
860	C-N
1037	C-O-C
1320	O-H (in phenol group)
1413	N-H
1615	O-H
1720	C=O
3385	O-H (in carboxyl acid group)
3300~3500	N-H
